# Letter to the Editor Regarding “Alterations of gut microbiome in chronic rhinosinusitis: insights from a mendelian randomization study”

**DOI:** 10.1016/j.bjorl.2026.101783

**Published:** 2026-03-10

**Authors:** Rafael Gomes de Melo D’Elia, Angélica Saiuri de Aurélio Penteado

**Affiliations:** Hospital das Clínicas da Faculdade de Medicina da Universidade de São Paulo (HCFMUSP), São Paulo, SP, Brazil

Dear Editor,

The article “Alterations of gut microbiome in chronic rhinosinusitis: insights from a Mendelian randomization study”^1^ represents an important and timely contribution to the expanding literature on the gut-airway axis. By applying a large-scale, bidirectional Mendelian Randomization (MR) design, the authors expand the analysis beyond small observational studies and provide valuable causal hypotheses regarding systemic consequences of Chronic Rhinosinusitis (CRS).

The use of MR is methodologically appropriate in this setting, particularly since there are powerful confounders, such as age, antibiotic exposure, and comorbidities, in microbiome research. However, it is important for clinicians to recognize that MR estimates lifelong genetic liability rather than short-term biological effects, and therefore cannot fully explain the underlying mechanisms linking CRS to gut microbial changes.^2^

Although the authors applied multiple sensitivity analyses to address pleiotropy ‒ including MR-Egger regression, weighted median estimation, Cochran’s *Q* testing, and leave-one-out analysis ‒ residual pleiotropy remains a relevant concern in microbiome MR studies. Genetic variants associated with microbial traits often influence immune and metabolic pathways directly, which may not be fully captured by standard statistical tests. Thus, the absence of detected pleiotropy should be viewed as reassuring but not conclusive.^3^

Another key limitation concerns functional interpretation. The reported alterations in microbial metabolic pathways are inferred from predicted gene content rather than direct metabolomic measurements. As a result, conclusions regarding reduced microbial metabolic activity or altered host-microbe interactions remain hypothetical and require validation through targeted metabolomics or experimental models.^4^

Additionally, CRS was analyzed as a single, homogeneous phenotype. Given the well-established biological differences between CRS endotypes^5^ ‒ particularly CRS with and without nasal polyps ‒ and the frequent use of antibiotics and corticosteroids, the lack of phenotypic or treatment stratification may obscure subtype-specific causal effects.

Finally, the restriction to individuals of European ancestry limits generalizability, and replication in more diverse populations will be essential. Therapeutic implications, including microbiota-targeted interventions, should therefore be considered exploratory at this stage. In summary, this study provides a robust, hypothesis-generating framework for understanding systemic microbiome alterations in CRS. Addressing these key limitations in future work will be crucial for translating MR-based findings into biological insight and clinical relevance for otorhinolaryngology practice.

Thank you for considering our thoughts on this important topic.

## ORCID ID

Angélica Saiuri de Aurelio Penteado: 0000-0001-5870-1828

## Data availability statement

The authors declare that all data are available in repository.

## Declaration of competing interest

The authors declare that the article content was composed in the absence of any commercial or financial relationships that could be construed as a potential conflict of interest.
